# Genetic mutations in Chinese patients with steatocystoma multiplex and mechanistic investigation of *KRT17* p.Arg94Cys

**DOI:** 10.1016/j.jdin.2026.05.017

**Published:** 2026-05-26

**Authors:** Jiachen Sun, Lihua Zhang, Jie Li, Hailong Chen, Chunlei Zhang, Chuan Ma

**Affiliations:** a Department of Dermatology, Peking University Third Hospital, Beijing, China; b Department of Pathology, Fourth Medical Center of Chinese PLA General Hospital, Beijing, China; c Department of Pathology, First Medical Center of Chinese PLA General Hospital, Beijing, China

**Keywords:** apoptosis resistance, KRT17 mutation, steatocystoma multiplex

*To the Editor:* Steatocystoma multiplex (SM) is a rare autosomal dominant genodermatosis characterized by multiple sebum-filled cysts arising from pilosebaceous units. Nonsynonymous mutations in *KRT17* are the best-established genetic cause of familial SM.^1^ However, the underlying molecular mechanisms remain poorly understood. Here, we report the clinical and genetic features of a Chinese SM cohort and provide preliminary mechanistic insights into the recurrent KRT17 p.Arg94Cys variant.

Nine Han Chinese patients (8 male, 1 female) were diagnosed with SM at our institution between January 2023 and December 2024 (Table I, Supplementary Fig 1, available via Mendeley at https://data.mendeley.com/datasets/74x5fgxmss/1). Cases 1-7 had a paternal family history. Age at onset ranged from 1 to 21 years (mean 14.3 years). Lesions predominantly involved the trunk and upper extremities. None had nail dystrophy. Genetic testing identified 2 known *KRT17* variants: c.280C>T (p.Arg94Cys, Supplementary Fig 2, available via Mendeley at https://data.mendeley.com/datasets/74x5fgxmss/1) in Case 1 and c.100G>A (p.Gly34Ser) in Case 2. WES additionally identified variants in *KRT2*, *GJB2*, *MVD*/*CYP27A1*, and *GNPTAB* (Cases 3-9), supported by multiple in silico predictions (ClinVar, SIFT, PolyPhen-2, MutationTaster, CADD Phred >15); all are classified as VUS pending segregation, ACMG classification, and functional validation.

**Table I:** Clinical and genetic characteristics of 9 patients in our cohort

Case	Sex/age	Family history	Age of onset	Affected sites	Mutation
1	M/12	Father	1	Whole body	*KRT17*, c.280C>T, p.Arg94Cys
2	M/33	Father	20	Trunk upper extremities	*KRT17*, c.100G>A, p.Gly34Ser
3	M/23	Father	11	Trunk	*KRT2*, c.300_301insGGCTTTGGAGGCGGC (p.Ser100_Ser101insGlyPheGlyGlyGly)
4	M/31	Father	15	Trunk upper extremities	*GJB2*, c.79G>A, p.Val27Ile
5	M/25	Father	14	Trunk upper extremities	*GJB2*, c.109G>A, p.Val37Ile
6	M/24	Father	21	Neck	*GJB2*, c.79G>A, p.Val27Ile *GJB2*, c.341A>G, p.Glu114Gly
7	M26	Father	15	Trunk	*GJB2*, c.109G>A, p.Val37Ile
8	M/25	-	18	Trunk upper extremities face	*MVD*, c.1085C>T, p.Pro362Leu *CYP27A1*, c.1420C>T, p.Arg474Trp
9	F/27	-	14	Upper extremities	*GNPTAB*, c.99del, p.Ala34ProfsTer49

We searched PubMed, Embase, and CNKI (January 2003-December 2023) using terms “steatocystoma multiplex,” “multiple steatocystoma,” and “steatocystomatosis” including only genetically or histopathologically confirmed SM cases with available clinical data. Among 145 identified cases (Supplementary Table I, available via Mendeley at https://data.mendeley.com/datasets/74x5fgxmss/1), *KRT17* was the predominant gene, with 13 distinct variants in 18 patients (Supplementary Table II, available via Mendeley at https://data.mendeley.com/datasets/74x5fgxmss/1). The most frequent hotspot was Arg94, including p.Arg94Cys (*n* = 5), p.Arg94His (*n* = 2), and p.Arg94Gly (*n* = 1). Notably, 3 patients with *KRT17* mutations also had KRT17-pEDD-PC, with mutations p.Met88Thr, p.Asn92Ser, and p.Leu99Pro, all within the Coil 1A domain.^2^

Given the recurrence of p.Arg94Cys and its identification in Case 1, we preliminarily investigated its molecular mechanism. KRT17 lacks a distinct drug-binding pocket, making direct therapeutic targeting difficult; we thus focused on protein interactions. AlphaFold modeling and STRING predicted that p.Arg94Cys substantially strengthens KRT17–SFN (14-3-3σ) binding, reducing predicted dissociation constants from 1.6 μM to 0.13 μM (Supplementary Fig 3, available via Mendeley at https://data.mendeley.com/datasets/74x5fgxmss/1). Immunoprecipitation in KRT17-knockout HeLa cells confirmed significantly enhanced KRT17–SFN interaction in p.Arg94Cys versus wild-type cells (Fig 1, *A*-*C*). Further modeling and immunoprecipitation demonstrated that this tighter complex promotes KRT17/SFN/BAD ternary complex formation, potentially sequestering BAD and impairing its proapoptotic function (Fig 1, *D* and *E*).^3^, ^4^, ^5^ TUNEL staining of clinical specimens showed markedly reduced apoptosis in SM cyst walls (0.01% to 0.09%) compared with solitary lipoma controls (3.48% to 7.65%), with low Ki-67 labeling (<1%), suggesting that apoptosis suppression rather than increased proliferation may drive cyst persistence (Fig 1, *F*). No published clinical studies have directly investigated apoptotic markers in SM. We acknowledge that this mechanistic model is based on *in vitro* findings, and direct correlation with clinical severity has not been established.

**Fig 1:**
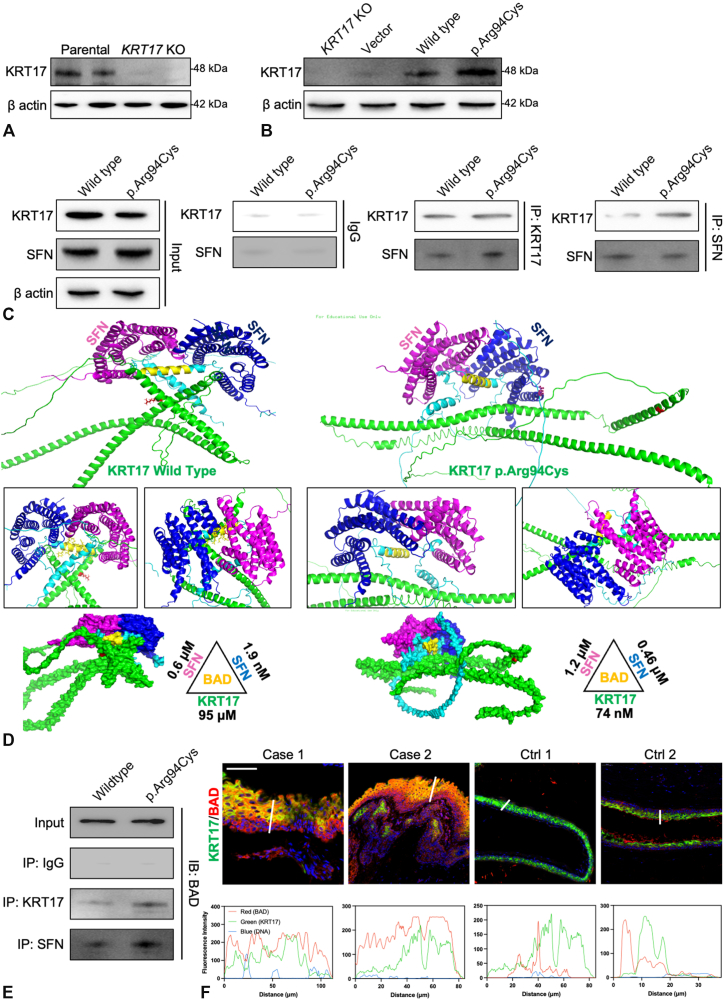
Impact of KRT17 p.Arg94Cys mutation on interactions with SFN and BAD. **A,** The efficacy of CRISPR/Cas9-mediated *KRT17* knockout in HeLa cells was confirmed by Western blot. *N* = 3. **B,** Western blot analysis of KRT17 in HeLa cells transfected with wild-type and variant *KRT17* cDNA constructs. *N* = 3. **C,** Co-immunoprecipitation was employed to detect interactions between KRT17 and SFN. *N* = 3. **D,** AlphaFold was leveraged to predict the formation of the KRT17-BAD-SFN protein complex and to assess the impact of the KRT17 p.Arg94Cys mutation on the structural dynamics of this complex. The architecture of KRT17 was delineated in green, with the 94th residue rendered in “stick” representation and highlighted in red. The dual subunits of SFN were distinctly color-coded in blue and magenta. BAD was depicted in cyan, with its pro-apoptotic BH3 motif distinctly marked in yellow. The KRT17-BAD-SFN complex was visually dissected from 3 perspectives, complemented by a surface representation depicted below. Additionally, a schematic triangular representation simplified the complex’s structure, with the K_d_ for BAD’s interaction with each protein numerically annotated on the corresponding edges. **E,** Co-immunoprecipitation assays were executed to delineate the interactive dynamics between KRT17-BAD and SFN-BAD complexes on HeLa cells. *N* = 3. **F,** Immunofluorescence staining of clinical specimens showing colocalization of KRT17 (green) and BAD (red). Line profiles below each image demonstrate the fluorescence intensity distribution and colocalization patterns. Scale bar = 100 μm.

In summary, we present a Chinese SM cohort with diverse genetic findings, highlighting the need for larger genotype–phenotype studies with segregation and functional validation. We provide preliminary evidence that KRT17 p.Arg94Cys may promote SM pathogenesis by enhancing KRT17/SFN/BAD complex formation and suppressing apoptosis, suggesting potential therapeutic targets warranting further investigation.

## Conflicts of interest

None disclosed.

## References

[1] Koprulu M., Naeem M., Nalbant G. (2022). KERATIN 17-related recessive atypical pachyonychia congenita with variable hair and tooth anomalies. Eur J Hum Genet.

[2] Ho M., Thompson B., Fisk J.N. (2022). Update of the keratin gene family: evolution, tissue-specific expression patterns, and relevance to clinical disorders. Hum Genomics.

[3] Tong X., Coulombe P.A. (2006). Keratin 17 modulates hair follicle cycling in a TNFalpha-dependent fashion. Genes Dev.

[4] Sun J., Zhao H., Shen C. (2022). Tideglusib promotes wound healing in aged skin by activating PI3K/Akt pathway. Stem Cell Res Ther.

[5] Pozuelo-Rubio M. (2010). Proteomic and biochemical analysis of 14-3-3-binding proteins during C2-ceramide-induced apoptosis. FEBS J.

